# Xylitol production from waste xylose mother liquor containing miscellaneous sugars and inhibitors: one-pot biotransformation by *Candida tropicalis* and recombinant *Bacillus subtilis*

**DOI:** 10.1186/s12934-016-0480-0

**Published:** 2016-05-16

**Authors:** Hengwei Wang, Lijuan Li, Lebin Zhang, Jin An, Hairong Cheng, Zixin Deng

**Affiliations:** Innovation and Application Institute (IAI), Zhejiang Ocean University, Zhoushan, 316022 China; State Key Laboratory of Microbial Metabolism, School of Life Sciences and Biotechnology, Shanghai Jiao Tong University, Shanghai, 200240 China

**Keywords:** Waste xylose mother liquor, One-pot biotransformation, Xylitol, *Candida tropicalis*, *Bacillus subtilis*

## Abstract

**Background:**

The process of industrial xylitol production is a massive source of organic pollutants, such as waste xylose mother liquor (WXML), a viscous reddish-brown liquid. Currently, WXML is difficult to reuse due to its miscellaneous low-cost sugars, high content of inhibitors and complex composition. WXML, as an organic pollutant of hemicellulosic hydrolysates, accumulates and has become an issue of industrial concern in China. Previous studies have focused only on the catalysis of xylose in the hydrolysates into xylitol using one strain, without considering the removal of other miscellaneous sugars, thus creating an obstacle to subsequent large-scale purification. In the present study, we aimed to develop a simple one-pot biotransformation to produce high-purity xylitol from WXML to improve its economic value.

**Results:**

In the present study, we developed a procedure to produce xylitol from WXML, which combines detoxification, biotransformation and removal of by-product sugars (purification) in one bioreactor using two complementary strains, *Candida tropicalis* X828 and *Bacillus subtilis* Bs12. At the first stage of micro-aerobic biotransformation, the yeast cells were allowed to grow and metabolized glucose and the inhibitors furfural and hydroxymethyl furfural (HMF), and converted xylose into xylitol. At the second stage of aerobic biotransformation, *B. subtilis* Bs12 was activated and depleted the by-product sugars. The one-pot process was successfully scaled up from shake flasks to 5, 150 L and 30 m^3^ bioreactors. Approximately 95 g/L of pure xylitol could be obtained from the medium containing 400 g/L of WXML at a yield of 0.75 g/g xylose consumed, and the by-product sugars glucose, l-arabinose and galactose were depleted simultaneously.

**Conclusions:**

Our results demonstrate that the one-pot procedure is a viable option for the industrial application of WXML to produce value-added chemicals. The integration of complementary strains in the biotransformation of hemicellulosic hydrolysates is efficient under optimized conditions. Moreover, our study of one-pot biotransformation also provides useful information on the combination of biotechnological processes for the biotransformation of other compounds.

## Background

Commercially available xylitol is mainly produced by the hydrogenation of xylose under conditions of high temperature (80–140 °C) and high pressure (up to 50 atm) [1–4]. The intermediate sugar xylose is extracted from hydrolysates of corn cobs and sugarcane bagasse through acid hydrolysis, condensation and crystallization. These processes are massive sources of organic pollutants such as waste xylose mother liquor (WXML), a viscous reddish-brown liquid. WXML mainly contains sugars (in w/w), such as xylose (35–40 %), l-arabinose (10–15 %), glucose (8–10 %) and galactose (8–10 %), and other organic components such as furfural and hydroxymethyl furfural (HMF), the concentrations of which depend on the factory as well as different batches. Generally, WXML contains approximately 60–75 % (in w/w) total sugars, among which xylose accounts for 50–70 % [5]. However, xylose, the most prevalent sugar in WXML, cannot be extracted by crystallization due to the high content of other sugars. Currently, WXML is difficult to reuse due to its miscellaneous low-cost sugars, high content of inhibitors and complex composition. In China, 50,000–80,000 tons of WXML is estimated to be produced by about ten large factories per year, and this has become an issue of industrial concern.

**Fig. 1:**
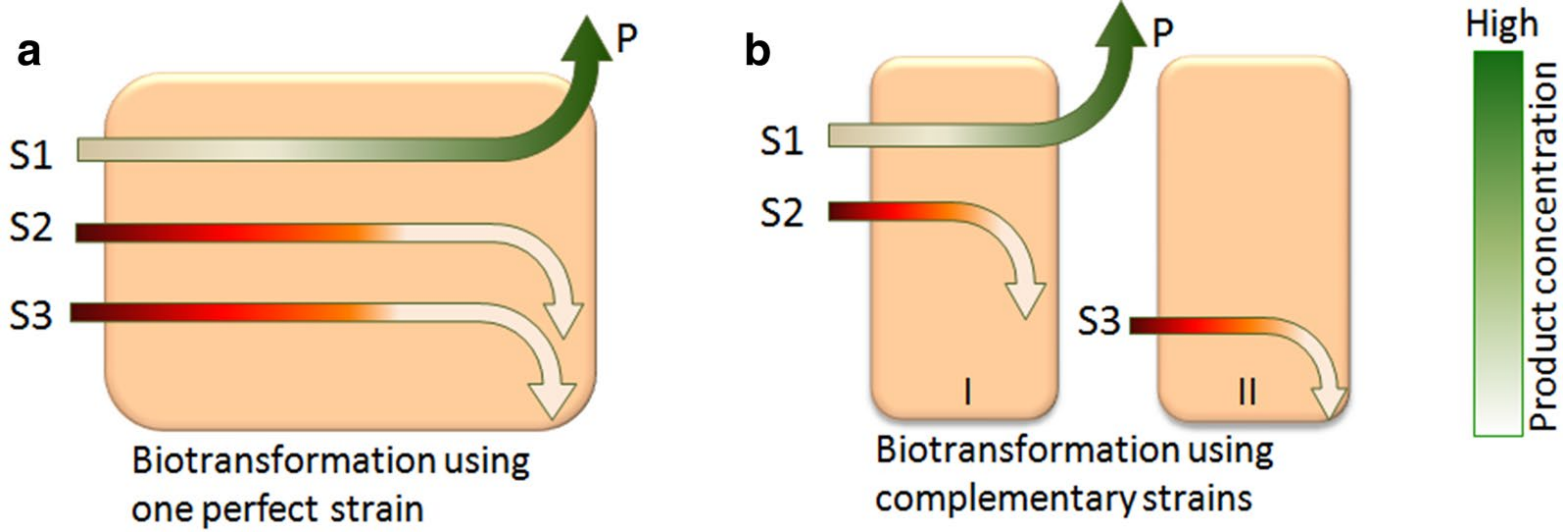
Scheme of biotransformation by one perfect strain (**a**) and complementary strains (**b**). *S1* main substrate xylose, *S2* by-product sugars, *S3* inhibitors, *P* product, *I* and *II* complementary strains

Attempts have been made to improve the economic value of WXML so as to facilitate its industrial application. One strategy is to individually separate the sugars, such as xylose, l-arabinose and galactose, by the methods of simulated moving bed chromatography and ion exchange chromatography [6, 7]. However, until now, the chromatography method is difficult to scale up in factories, due to its high running costs compared with the low-value sugars as well as its low separation efficiency. Another strategy is to directly transform the sugars, mainly xylose from hemicellulosic hydrolysates, into xylitol by yeast strains or other microbes [8–16]. It has been reported that approximately 100 g/L of xylitol can be produced from detoxified horticultural waste hemicellulosic hydrolysates using *Candida athensensis* SB18, a yeast strain isolated from soil [17]. The furan compounds furfural and HMF, released from dilute acid hydrolysis under severe conditions, are toxic to microorganisms used for the subsequent biotransformation. Treatment with 2–5 % (w/v) activated charcoal is a classic method to remove such growth inhibitors, but recently developed biological detoxification (biodetoxification) has shown potential in industrial applications due to its low cost [5, 18–20]. However, it is still difficult to scale up, since a high content of by-product sugars which are left in the biotransformation can significantly reduce the recovery of subsequent xylitol extraction [21–23]. There are two options to removal such by-product sugars by biochemical approaches (Fig. 1a, b). The first scheme is to use one “perfect” strain that can transform xylose to xylitol and consume all of the by-product sugars under the stress of inhibitors (Fig. 1a). The alternative is to use “complementary” strains, one of which could either transform xylose to xylitol, or consume by-product sugars or detoxify the inhibitors (Fig. 1b). The first one seems very simple in terms of processing, but it is sometimes quite difficult to construct such a “perfect” strain. In our previous study, we developed a technical route in which biodetoxification, biotransformation and purification was integrated using *C. maltosa* and recombinant *B. subtilis* with a disrupted xylose isomerase gene *xyl*A. We finally obtained 213 g/L of xylitol in a medium containing 250 g/L of xylose from WXML in a 5 L fermentor [5]. However, it needs improvements due to the complexity of the technique. Specifically, centrifugation to clear the broth was necessary following each step so as to carry out next step of catalysis; the use of *C. maltosa* produced approximately 5 g/L of d-arabitol from xylose, thereby reducing the yield of xylitol; and before the last step of xylose biotransformation, vacuum evaporation was performed to concentrate the fermentation broth to obtain 250 g/L of xylose. This technology is still not suitable for simple large-scale xylitol production from WXML due to its complicated operation and considerable equipment investment.

If pure xylitol can be produced directly from the microbial fermentation of WMXL or hemicellulosic hydrolysates using only one bioreactor, and the inhibitors and by-product sugars can be removed simultaneously, the technique might be simple and competitive enough to be industrialized. In the present study, we aimed to develop a one-pot procedure to produce xylitol from WXML, in which detoxification, biotransformation and purification were carried out in only one bioreactor. To achieve this purpose, we first constructed an integrated biotransformation system using two complementary strains. Secondly, we tested its integration efficiency, optimized the conditions, and developed a two-stage biotransformation, which transformed xylose into xylitol without producing new sugar alcohols, and meanwhile, depleted the inhibitors and by-product sugars. Finally, we successfully scaled up our newly developed one-pot biotransformation from shake flasks to 150 L and 30 m^3^ bioreactors, and its advantages were discussed. Our technical strategy may be helpful in the production of other chemicals from hemicellulosic hydrolysates.

## Results and discussion

### Screening and characterization of target yeast strains

**Fig. 2:**
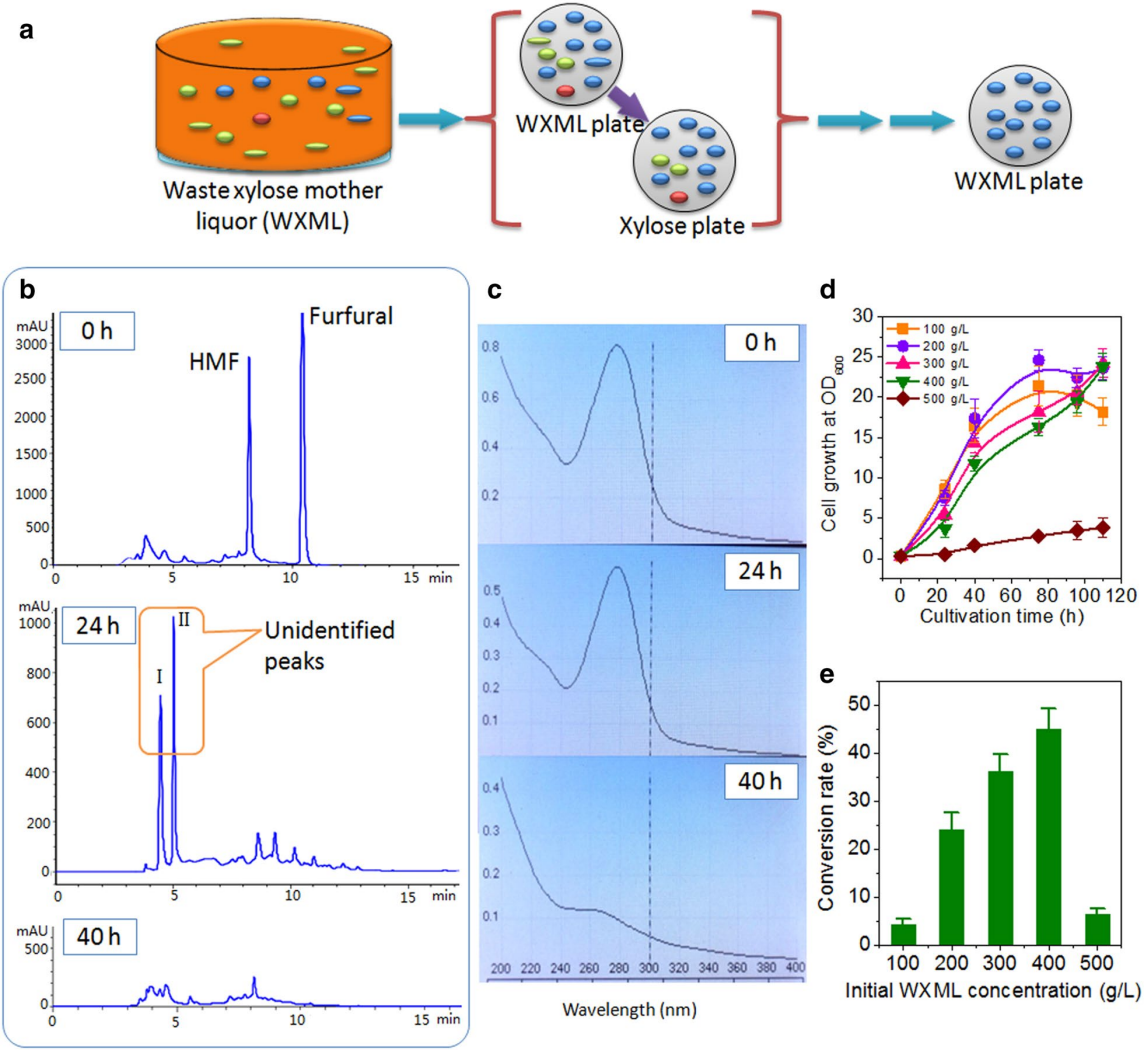
Screening and characterization of target yeast strain *C. tropicalis* X828. **a** Screening of yeast strains from WXML using WXML plates and xylose plates; **b** HPLC profiles of WXML detoxification by *C. tropicalis* X828 at 0, 24 and 40 h; *I*, *II* unidentified peaks; **c** measurement of A_280_ as an indicator of the degradation of furfural and HMF; **d** effects of initial WXML concentration on yeast cell growth; **e** effects of initial WXML concentration on the xylose conversion rate (%). The shake flask experiments were performed in duplicate, and the results were expressed in the form of average value ± standard deviation

WXML contains approximately 800 g/L of total sugars and 5–10 g/L of furan compounds (mainly furfural and HMF), with a density of 1.25 g/L and a low pH at 3.5–4.5, thus presenting a harsh environment for microbial survival. In general, longer storage time would lead to more yeast strains enriched in the WXML samples. In the case of yeast strain screening, we used samples from WXML stored for several years in factories rather than fresh WXML. This may help to collect the yeast strains that can survive in WXML containing high concentrations of inhibitors. Only the strains that grew well on both WXML plates and xylose plates were collected and further cultivated for at least 20 rounds on WXML plates for adaption (Fig. 2a). Following the above procedure, we chose one from several candidates and designated it as *C. tropicalis* X828 based on the sequences of its 18S rDNA (99 % in homology test) and internal transcribed spacer (ITS) region (100 % in homology test).

The detoxification of WXML was performed in a complex YCN medium (complex medium in which nitrogen sources included yeast extract, corn syrup powder and (NH_4_)_2_HPO_4_) containing 400 g/L WXML and the concentration of furfural and HMF was estimated at 2.1 and 1.5 g/L. Analysis by high-performance liquid chromatography (HPLC) and measurement of biotransformation aliquots at their peak absorbance at 280 nm (A_280_) demonstrated that furfural and HMF could be depleted in about 40 h (Fig. 2b, c). The results show that the detoxification capacity of *C. tropicalis* X828 met the requirements for further experiments; the initial cell density we used was only 0.6 at OD_600_. Additionally, our HPLC results also showed the presence and disappearance of two unidentified peaks (I, II) when yeast cells entered the exponential growth phase in about 24 h (Fig. 2b). Recently, it has been highlighted that *S. cerevisiae* strains have the ability to reduce furfural and HMF to their alcohols 2-furanmethanol (FM) and furan-2,5-dimethanol (FDM) [24–27]; two enzymes, alcohol dehydrogenase 6 (ADH6) and alcohol dehydrogenase 1 mutant (mut-ADH1), have been identified as the enzymes responsible for the reduction of furfural and HMF in *S. cerevisiae* [28, 29]. Almeida et al. [30] found that xylose reductase from *Pichia stipitis* (Ps-XR) is able to reduce HMF to its alcohol both in vitro and in vivo and suggested that Ps-XR also has furaldehyde reduction abilities. It has been suggested that the accumulation of these metabolites may be less toxic to yeast cells than their aldehyde forms [29, 31]. Based on the above reports, the unidentified peaks were speculated to be their alcohol compounds, and this needs further investigation.

The strain *C. tropicalis* X828 grew very well and formed large colonies (2–3 mm in diameter) within approximately 60 h on solid plates containing 500 g/L WXML; however, in the liquid YCN medium containing WXML at the same concentration, the cell growth almost stopped and the cell concentration reached only 1–2 at OD_600_ in 48 h (Fig. 2d). In contrast, the initial concentrations of WXML ranging from 100 to 400 g/L supported its growth well and biomass of more than 23 at OD_600_ could be achieved at the end of cultivation (Fig. 2d). Correspondingly, the conversion rate of xylose was also decreased in the presence of 500 g/L WXML due to poor biomass formation (Fig. 2e). Additionally, fewer sugars in the medium, i.e. a content of 100 g/L WXML, also decreased the xylose conversion rate (Fig. 2e). In fact, under the conditions of 100 g/L WXML, other sugars such as glucose, galactose but l-arabinose were also exhausted at the end of cultivation due to the metabolic demands of cellular growth (data now shown). The maximum yield of xylitol, approximately 46 %, was observed only when using 400 g/L WXML (Fig. 2e). Through the biodetoxification by yeast cells, the 127 g/L of xylose from 400 g/L WXML was converted into 59 g/L of xylitol, the glucose (13.5 g/L) was exhausted, and the two by-product sugars l-arabinose (45.2 g/L) and galactose (11 g/L), remained.

### Screening of *B. subtilis* strains and sugar metabolism by complementary strain Bs12

**Fig. 3:**
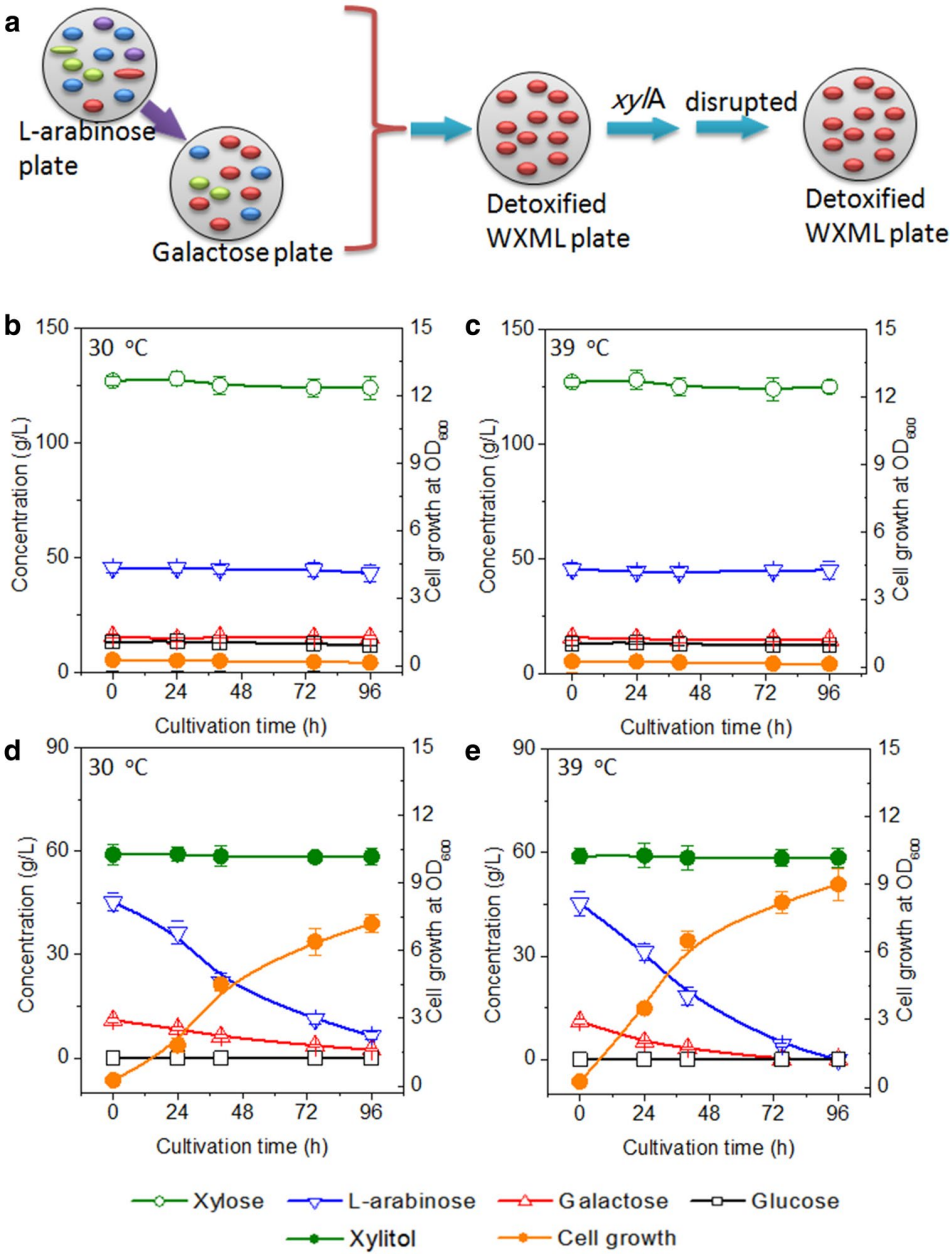
Screening and characterization of complementary strain *B. subtilis* Bs12. **a** Screening of complementary strains for the target yeast strain using l-arabinose or galactose containing NSM plates. The complementary strain was then cultivated for 20–30 rounds on detoxified WXML plates. *xyl*A, xylose isomerase gene *xyl*A; Sugar metabolism of the complementary strain *B. subtilis* Bs12 is shown in medium containing undetoxified WXML at 30 °C (**b**) and 39 °C (**c**) and in detoxified WXML medium at 30 °C (**d**) and 39 °C (**e**). The shake flask experiments were performed in duplicate, and the results were expressed in the form of average value ± standard deviation

Results from plates showed that *C. tropicalis* X828 was poor in its capacity to deplete l-arabinose, which is the most prevalent by-product sugar in WXML. Thus, to complete the one-pot biotransformation complementary strains were necessary. *B. subtilis*, well known as an aerobe commonly found in soil, can metabolize a variety of sugars. The complementary strain should consume l-arabinose and galactose but not the final product xylitol (Fig. 3a). In our previous study, we have shown that the *xyl*A gene-disrupted *B. subtilis* 168 lose its ability to metabolize xylitol [5]. Thus, we decided to use the *xyl*A gene-disrupted *B. subtilis* 168 as a starter strain to further improve its adaptability on the detoxified WXML plates (Fig. 3a). Only the top five colonies that grew well on the plates were chosen and applied to the next round of adaption. Following the above procedure, we obtained a well-adapted strain and designated it as *B. subtilis* Bs12.

However, when cultivated in YCN medium containing 400 g/L of undetoxified WXML, strain Bs12 showed no signs of cell growth or sugar metabolism at 30 or 39 °C (Fig. 3b, c), but in YCN medium containing detoxified WXML, it grew to approximately 7.2 at OD_600_ within 96 h from an initial cell concentration of 0.25 at OD_600_ (Fig. 3d). At 30 °C, this strain degraded l-arabinose (45.2 g/L) and galactose (11 g/L) to approximately 6.5 and 2.4 g/L within 96 h (Fig. 3d). When the cultivation temperature was increased to 39 °C, its cell growth was improved and l-arabinose and galactose were completely depleted within 96 h (Fig. 3e). The concentration of xylitol remained constant throughout the cultivation, suggesting that *B. subtilis* Bs12 could not metabolize xylitol and thus it was suitable for subsequent purification experiments (Fig. 3e). These results suggest a promising scheme for the biotransformation of xylose from WXML and purification by the consumption of by-product sugars using two complementary strains.

### Complementary biotransformation of xylose from WXML by *C. tropicalis* X828 and *B. subtilis* Bs12 in shake flasks

**Fig. 4:**
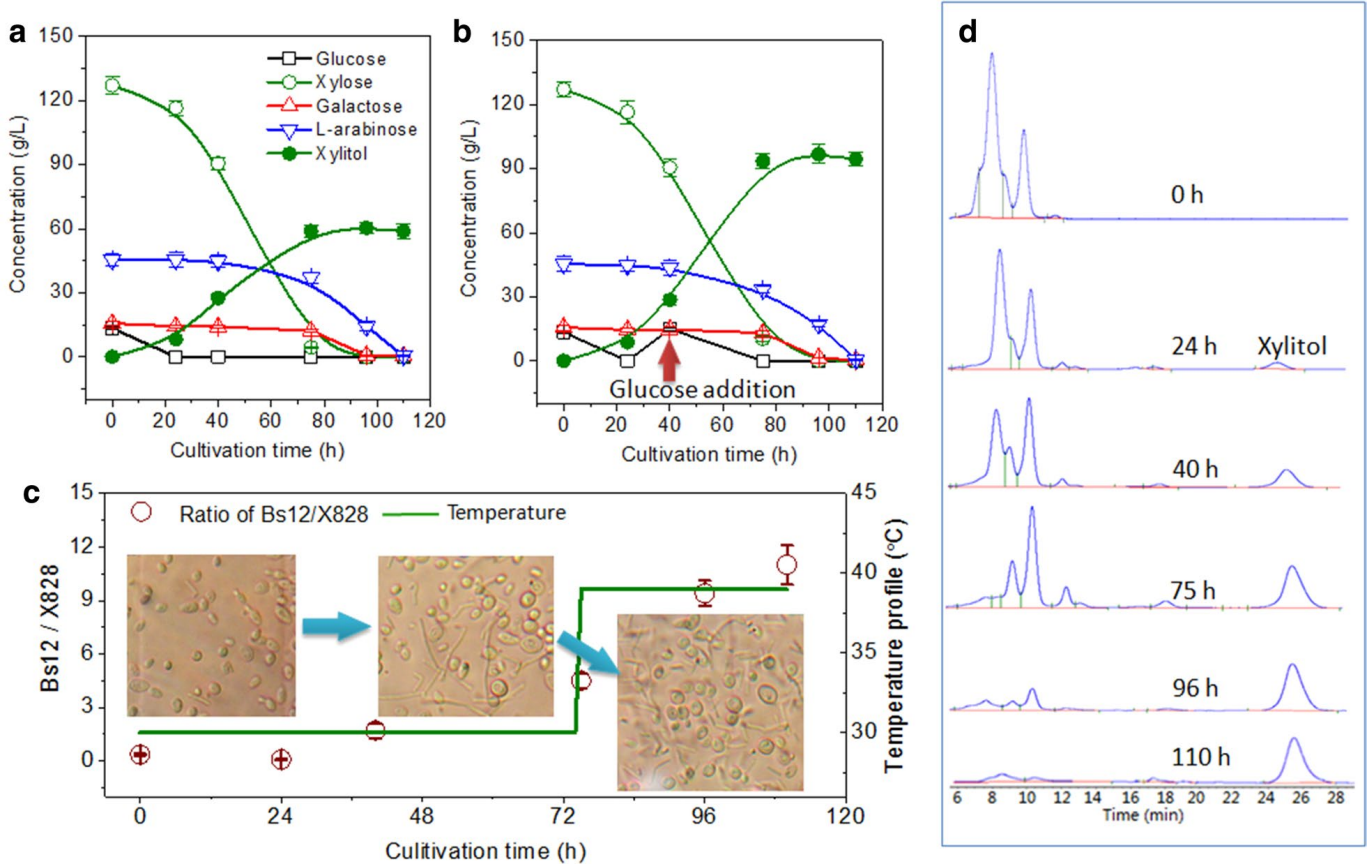
Complementary biotransformation of WXML by *C. tropicalis* X828 and *B. subtilis* Bs12 in shake flasks. **a**, **b** Batch biotransformation and glucose fed-batch biotransformation; the arrow represents the addition of 15 g/L glucose at 40 h; **c** ratio of strain Bs12 and X828 cells in glucose fed-batch biotransformation; **d** HPLC profiles of xylitol production. The shake flask experiments were performed in duplicate, and the results were expressed in the form of average value ± standard deviation

In the above experiments, we tested the existence of complementary metabolic pathways in the two strains on plates. To run a successful complementary transformation, we also needed to test their integration efficiency to make both match for each other in one bioreactor. In the experiments of complementary biotransformation, precultures of yeast strain X828 and *B. subtilis* Bs12 were inoculated at the same time into YCN medium containing 400 g/L of WXML. The 13.5 g/L of glucose in WXML was depleted first within about 24 h and the by-product sugars, 15.5 g/L of galactose and 45.2 g/L of l-arabinose, were exhausted at 96 h and 110 h, respectively (Fig. 4a). Additionally, a maximum concentration of 2.2 g/L of ethanol at 75 h and less than 1 g/L of arabitol at 110 h were also observed. At the end of biotransformation, only 59 g/L of xylitol was obtained from 127 g/L of xylose, and the conversion rate was estimated at approximately 46 % (Fig. 4a). In fact, the maximum yield of xylitol was observed at approximately 80–96 h, but at that moment, a total of 15 g/L of by-product sugars was still left in the medium.

The addition of 15 g/L glucose at 40 h, following the depletion of the initial glucose from WXML, increased the final production of xylitol to 94.5 g/L and the final conversion rate reached 73 % (Fig. 4b). When there was low content of sugars from WXML, for example, 100 g/L of WXML (Fig. 2e), most of the sugars including xylose would be depleted at the end of cultivation, suggesting that a sufficient amount of glucose was indeed required to avoid xylose consumption for cellular growth. Similar results from Yahashi et al. [32] also suggested that yeast cells consume xylose for the production of xylitol as well as for cell growth and maintenance, thus resulting in decreased xylitol yield. However, in the case of xylitol production by a recombinant *S. cerevisiae* strain containing a xylose reductase gene from *Pichia stipitis*, supplemented glucose above 0.35 g/L inhibited xylose uptake and caused ethanol accumulation up to 48 g/L [33]. This suggests that the choice of yeast strain, xylose content as well as glucose supplementation are crucial to obtain a high yield of xylitol.

Figure 4c shows that the initial ratio of Bs12/X828 cell concentrations was approximately 0.36 at 0 h and decreased to 0.08 at 24 h due to the cell growth of yeast strain X828 under the stress of the inhibitors furfural and HMF. After 48 h, *B. subtilis* strain Bs12 outgrew yeast cells and continued to grow to a final ratio of 11. The growth improvement of strain Bs12 was mainly due to the depletion of furaldehyde inhibitors by yeast strain X828 and an increase in temperature to 39 °C at 72 h. From 75 to 110 h, the maximum consumption rates of galactose and l-arabinose reached 0.5 and 1.0 g/(L·h) with the fast growth of strain Bs12 (Fig. 4b, c). Figure 4d demonstrates the HPLC profiles of xylitol production and the consumption of by-product sugars. Especially during the aerobic period (75–110 h), approximately 80 % of all the by-product sugars were consumed (Fig. 4b, d). At the end of biotransformation, the by-product sugars were depleted to less than 4 g/L in total concentration, and the purity of xylitol produced was estimated at 95 %. The biomass produced was approximately 3.2 g/L dry cell weight (DCW) thus facilitating the subsequent xylitol extraction procedure by simply removing the cells.

### Complementary biotransformation of xylose from WXML in bioreactors

**Fig. 5:**
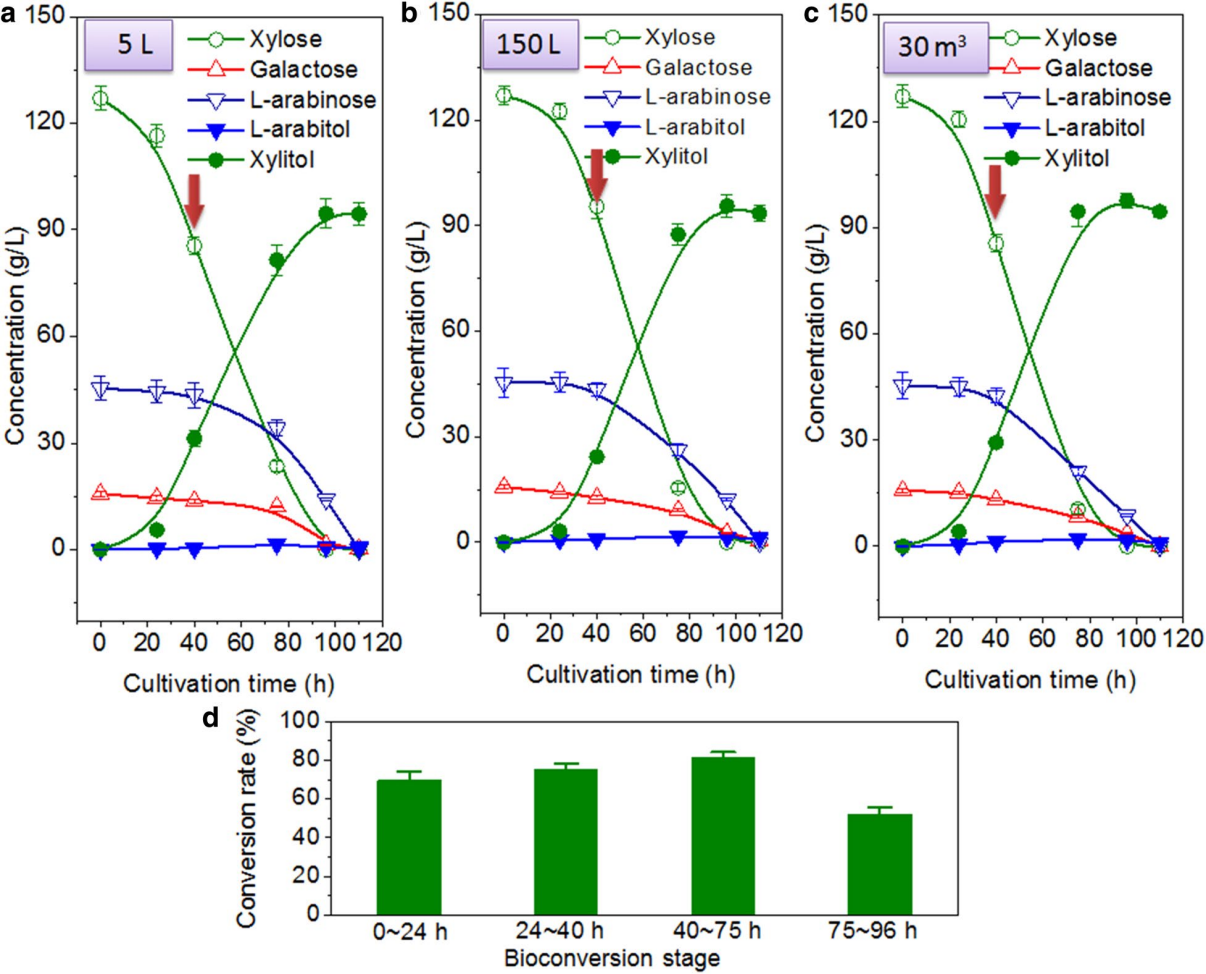
Complementary biotransformation of WXML by *C. tropicalis* X828 and *B. subtilis* Bs12 in bioreactors. Results from 5 L (**a**), 150 L (**b**), and 30 m^3^ (**c**) bioreactors are shown; the *arrow* represents the addition of 15 g/L glucose at 40 h; the profiles of biotransformation conducted in bioreactors were from one batch of cultivation and expressed in the form of average value ± standard deviation. **d** Xylose conversion rates at different growth stages in a 5 L bioreactor. The results were from three batches of cultivation in a 5 L bioreactor

Based on the above results from shake flasks, we summed up the operating points: one-off inoculation, two-stage biotransformation and glucose supplementation. The batch biotransformation experiment in bioreactors was first performed on the scale of 5 L (Fig. 5a). At the first stage of micro-aerobic biotransformation (0–72 h), the yeast cells were allowed to grow and metabolized the inhibitors furfural and HMF, and approximately 82 % of the initial xylose (initial concentration = 127 g/L) was consumed/transformed and 82 g/L of xylitol was produced, while only 25 % of l-arabinose (initial concentration = 45.4 g/L) and 22 % of galactose (initial concentration = 15.3 g/L) was depleted. In contrast, the 13.5 g/L of initial glucose from WXML was exhausted as early as 24 h (data not shown), and more glucose (15 g/L) was then supplemented at 40 h to allow the transformation to continue into the second stage. At the second stage of aerobic biotransformation (72–110 h), with the fast cell growth of strain Bs12 the consumption rates of l-arabinose and galactose increased, and the rest of l-arabinose (34 g/L) and galactose (12 g/L) were depleted at approximately 96–110 h (Fig. 5a). At the end of biotransformation, 95 g/L of xylitol was produced and the overall conversion rate was estimated to be approximately 74 % (Fig. 5a). This represented a yield of 82 % of the theoretical value, which was computed to be 0.74 mol xylitol per mol xylose. The profiles of xylose biotransformation preformed in the 150 L and 30 m^3^ bioreactors were similar to the results from the 5 L bioreactor (Fig. 5b, c).

The detailed conversion rates of xylose at different stages were from three batches of biotransformation conducted in a 5 L bioreactor (Fig. 5d). Figure 5d demonstrates that the maximum conversion rate (approximately 77 %) was from the period of 40–75 h when there was a large amount of xylose and sufficient glucose for cell metabolism (Fig. 5a). The conversion rate at 75–96 h dropped to 55 %, suggesting that further optimization might be helpful. The oxidation–reduction balance of pyridine nucleotide cofactor (NADP^+^) is of great concern in xylitol biotransformation. Barbosa et al. [34] showed that the maximum yield of xylitol was 0.905 mol xylitol per mol of xylose consumed if NADPH was completely regenerated via the pentose phosphate pathway. However, with cell growth, the xylitol yield should be less than the theoretical value, since some of xylose is always consumed for cellular metabolism. Yahashi et al. [32] suggested that if a fermentation process, such as the optimization of glucose feeding, is applied to prevent xylose consumption for cellular growth and NADPH regeneration, the yield should be further increased.

### Comparison of two processes in the biotransformation of xylose from WXML

**Fig. 6:**
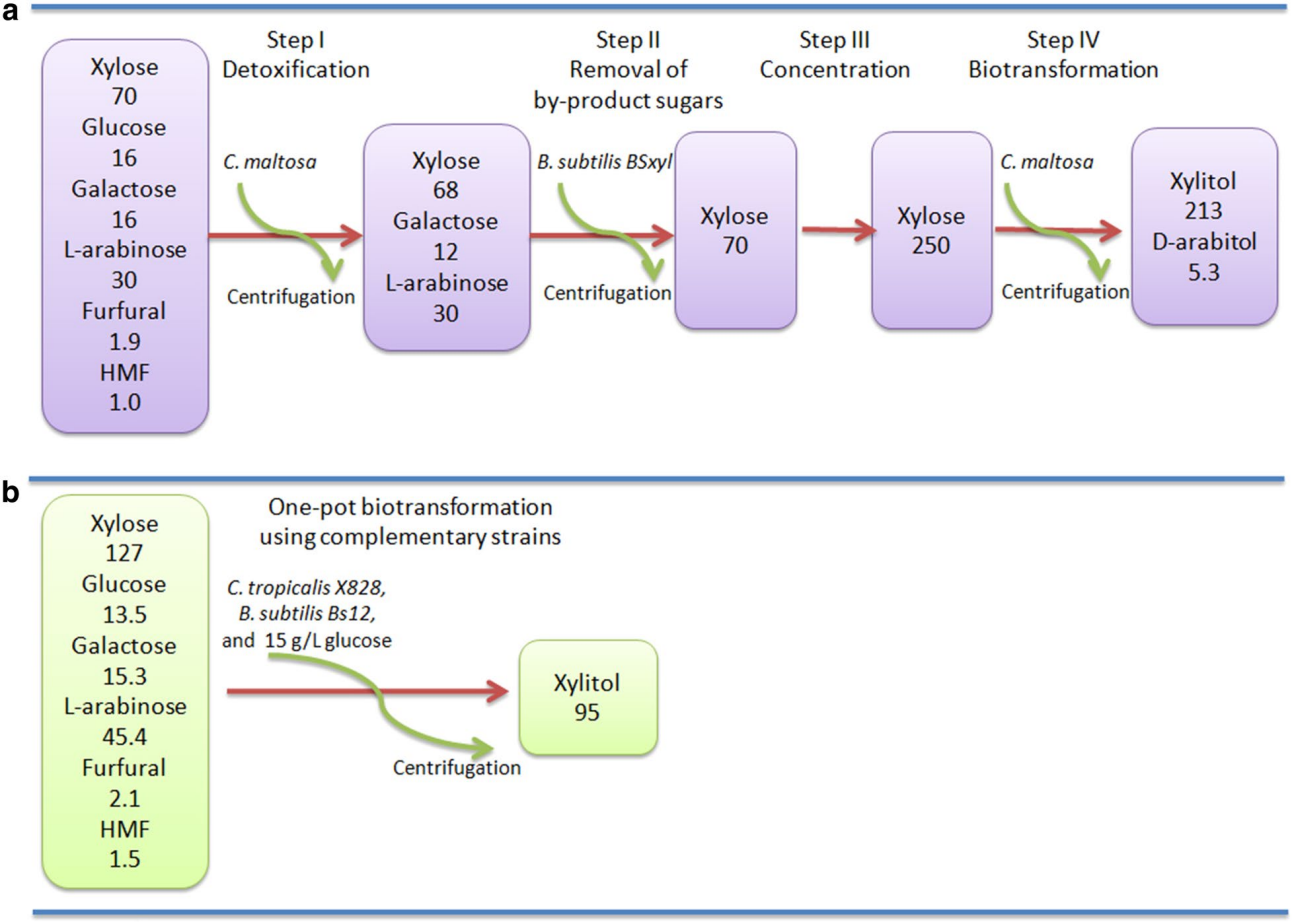
Comparison of two processes in the biotransformation of xylose from WXML. **a** Complementary biotransformation using *C. maltosa* and *B. subtilis* Bsxyl in our previous study; **b** one-pot biotransformation using *C. tropicalis* X828 and *B. subtilis* Bs12

Our group previously reported a method for the biotransformation of xylose from WXML, in which sugars and inhibitors were a little different from the WXML used in our present study [5]. In brief, there were four steps in our previous process: (i) Detoxification; the yeast strain *C. maltosa* was first inoculated into the medium containing WXML to remove the inhibitors and then collected by centrifugation. (ii) Purification; the *B. subtilis* strain Bsxyl was inoculated into the detoxified medium to deplete the by-product sugars l-arabinose and galactose and the cells was then removed by centrifugation. (iii) WXML concentration; the biopurified broth was condensed to obtain a higher content of xylose. (iv) Xylose biotransformation; the above collected yeast cells catalyzed xylose into xylitol in the concentrated broth (Fig. 6a). The conversion rate of xylose in step iv was estimated at 84 %, but the total one was only 63 % considering xylose loss in the cell precipitate during multi-step centrifugation and in the conversion into by-product d-arabitol. In the one-pot biotransformation, the yeast strain X828 and *B. subtilis* strain Bs12 were inoculated at the same time into WXML, and the detoxification, removal of by-product sugars, and xylose biotransformation were conducted in one bioreactor, following a two-stage cultivation procedure (Fig. 6b). The overall conversion rate in our newly developed one-pot biotransformation reached approximately 74 %. Additionally, our previous four-step biotransformation of xylose took about 10 days while the newly developed one-pot biotransformation took 110 h (Fig. 6).

Recently, there have been many papers published on xylitol production by the biotransformation of purified xylose and hemicellulosic hydrolysates, using one yeast strain obtained from screening or genetic modification. Kwon et al. [35] reported that they obtained 234 g/L xylitol form 260 g/L of pure xylose using *C. tropicalis* by fed-batch fermentation. Walther et al. studied the influence of hemicellulosic sugars on xylitol production by *C. tropicalis*, and demonstrated that the maximum xylitol yield (0.84 g/g xylose) was obtained using a synthetic medium containing high arabinose and low glucose and mannose contents [36]. However, the industrial production of xylitol is still carried out by chemical hydrogenation of pure xylose due to the cost of biotransformation in terms of industrial investment and processing time [1–3]. In recent years, xylitol production directly from hemicellulosic hydrolysates has made great progress. For instance, Ling et al. [37] optimized the culture medium in xylitol production by *C. tropicalis* HDY-02 and showed that 58 g/L of xylitol with a yield of 0.73 g/g xylose could be produced from corncob hydrolysates by fed-batch cultivation. Zhang et al. described the production of xylitol from horticultural waste hemicellulosic hydrolysates by a new strain of *Candida athensensis* SB18. This strain was able to consume up to 300 g/L of pure xylose at a yield of 0.83–0.87 g/g xylose and produced approximately 100 g/L of xylitol from hydrolysates that were detoxified by activated charcoal powder and concentrated using vacuum evaporation [17]. However, the above studies seldom report results from the consumption of by-product sugars, which are always produced in hemicellulosic hydrolysis. It has been reported that the crystallization of xylitol is negatively affected by the content of by-product sugars [22, 23]. Therefore, to achieve large-scale industrial production of xylitol directly from the biomass mixtures, the removal of by-product sugars should receive more consideration in future. In our present study, to removal such miscellaneous by-product sugars we chose to make use of the existing metabolic pathways in *B. subtilis*, rather than to construct new pathways in yeast cells. Our results demonstrate that the metabolic pathways borrowed are also efficient, in particular, in the biotransformation of mixtures of various ingredients.

## Conclusions

We report a one-pot strategy to make use of an organic pollutant of hemicellulosic hydrolysates WXML, which combined detoxification, biotransformation and purification in one bioreactor using two complementary strains, *C. tropicalis* X828 and *B. subtilis* Bs12. The one-pot biotransformation of WXML was successfully scaled up from shake flasks to 30 m^3^ bioreactors. Approximately 95 g/L of xylitol (95 % in purity) could be obtained from 400 g/L of WXML at a yield of 0.75 g/g xylose consumed, and the by-product sugars glucose, l-arabinose and galactose were depleted simultaneously. Our study provides useful information in the combination of biotechnological processes for the biotransformation of similar compounds.

## Methods

### Screening and identification of yeast strains

To prepare solid medium (WXML plates) for the screening of yeast strains, 100 mL of WXML from different factories (stored for more than 2 years) was sterilized separately at 110 °C for 30 min and mixed at 50–70 °C with 100 mL of sterilized YP medium containing 10 g/L yeast extract (Oxoid, UK), 5 g/L peptone (Oxoid, UK) and 25 g/L agar powder. Solid YP medium containing 20 g/L xylose (YPX) was also used for screening. Solid YP medium containing 20 g/L glucose (YPD) was used for the estimation of colony forming units (CFU) of yeast and *B. subtilis* cells.

To screen yeast strains that would grow well on WXML plates, the microbial cells were collected by centrifugation of 50 mL WXML at 10,000*g* for 20 min, re-suspended in sterile water, spread on WXML plates and cultivated at 30 °C for several days. For each round of cultivation, the top five largest yeast colonies were selected and subjected to the next cultivation step. In our experiments, most bacteria could not grow within 5 days on the WXML plates containing 500 g/L WXML.

Yeast strains were identified by the sequences of their 18S rDNA and ITS region, and genomic DNA was extracted using the phenol lysis method [38]. The primers used for amplification of 18S rDNA were 5′-ATC CTG CCA GTA GTC ATA TGC TTG TCT C-3′ and 5′-GAG GCC TCA CTA AGC CAT TCA ATC GGT A-3′ and those for ITS were 5′-TCC TCC GCT TAT TGA TAT GC-3′ and 5′-TTC GTA GGT GAA CCT GCG G-3′. PCR conditions were as follows: 94 °C for 5 min, 35 cycles of denaturation at 94 °C for 30 s, annealing at 55 °C for 30 s, extension at 72 °C for 90 s, and a final extension at 72 °C for 10 min. Purified PCR product was ligated into T-vector (pMD18, Takara, Dalian, China) and sequenced. The sequence analysis was conducted using the basic local alignment search tool (BLAST) from National Center for Biotechnology Information (NCBI).

### Screening and adaption of *B. subtilis* strains

The solid nitrogen and salts medium (NSM) used to screen *B. subtilis* strains contained 2 g/L (NH_4_)_2_SO_4_, 1 g/L casamino acids (AMRESCO), 0.5 g/L tryptophan, 5 g/L Na_2_HPO_4_·12H_2_O, 0.5 g/L KH_2_PO_4_, 0.2 g/L CaCl_2_, 0.5 g/L MgSO_4_·7H_2_O, 0.005 g/L MnCl_2_, 0.005 g/L FeSO_4_ and 15 g/L agar powder. To obtain a *B. subtilis* strain that could utilize l-arabinose and galactose but not xylose, all the *B. subtilis* strains from our laboratory stock were first screened on solid NSM plates containing 20 g/L l-arabinose or galactose, and then cultured at 37 °C for 20–30 rounds on solid NSM plates containing 200 g/L detoxified liquid WXML to further improve its adaptability. For each round of cultivation, the top five largest colonies were selected for their fast growth. After the above screening, we found the *xyl*A gene-disrupted *B. subtilis* 168 grew well on l-arabinose and galactose but not xylitol [5]. So we used the *xyl*A gene-disrupted *B. subtilis* 168 as a starter strain in the following adaption on detoxified WXML plates.

### Biotransformation and detoxification of WXML by yeast cells in shake flasks

The complex medium used for biotransformation (YCN medium) contained 5 g/L yeast extract (AngelYeast Co. Ltd, China), 5 g/L corn syrup powder, 2 g/L (NH_4_)_2_HPO_4_ and 0.5 g/L MgSO_4_·7H_2_O (pH 6.0). To prepare the yeast preculture, several colonies were inoculated into YCN medium containing 50 g/L glucose and cultivated at 30 °C for about 30 h. To start the biotransformation and detoxification, 1 mL of yeast preculture (OD_600_ = 30 ± 3) was inoculated to 50 mL of YCN medium containing 100, 200, 300, 400 and 500 g/L of WXML and cultivated at 30 °C for 110 h. All experiments were conducted in duplicate in 250 mL Erlenmeyer flasks on a 30 mm orbital shaker at 150 rpm. Cell growth was determined by measurement of the optical density (OD) at 600 nm (OD_600_). Sugars and sugar alcohols were analyzed by HPLC. Furfural and HMF were analyzed using both HPLC and absorbance at 280 nm [5]. The conversion rate of xylose (in %) was calculated by the formula of 98.7 × (g xylitol produced/g xylose consumed).

### Purification of WXML by *B. subtilis* cells in shake flasks

The purification experiments were performed in yeast-treated WXML using untreated WXML as control. The yeast-treated WXML was prepared as described in the above biotransformation and detoxification. In brief, 1 mL of yeast preculture was inoculated into the YCN medium containing 400 g/L WXML (supplemented with 15 g/L glucose at about 40 h) and cultivated for about 110 h. The medium was then centrifuged to remove yeast cells and sterilized at 110 °C for 20 min for purification experiments. The detoxified WXML medium contained approximate xylitol (59 g/L), l-arabinose (45.2 g/L) and galactose (11 g/L), but no glucose or inhibitors. To prepare the *B. subtilis* preculture, several colonies were inoculated to the YCN medium containing 20 g/L glucose and cultivated for about 24 h to reach 12 ± 2 at OD_600_. At inoculation, 1 mL of *B. subtilis* preculture was inoculated into 50 mL of the yeast-treated WXML and cultivated at 30 and 39 °C. All experiments were conducted in duplicate in 250 mL Erlenmeyer flasks on a 30 mm orbital shaker at 250 rpm.

### Complementary biotransformation of WXML in shake flasks

The precultures of yeast and *B. subtilis* were prepared as in the above biotransformation, detoxification and purification experiments. In brief, 1 mL of yeast precultures (OD_600_ = 30 ± 3) and 1 mL of *B. subtilis* precultures (OD_600_ = 12 ± 2) were inoculated at the same time into 50 mL of sterilized YCN medium containing 400 g/L of WXML. If necessary, 15 g/L of glucose was supplemented following the exhaustion of initial glucose. Fermentation conditions were set in two stages; in the first one, the temperature and shaking speed were 30 °C and 150 rpm, and in the second one, these parameters were changed to 39 °C and 250 rpm. The ratio of *B. subtilis* and yeast cells was estimated from their CFU on YPD agar plates. All the shake flask experiments were performed in duplicate, and the results were expressed in the form of average value ± standard deviation.

### Complementary biotransformation of WXML in bioreactors

Batch biotransformation of WXML in bioreactors was carried out in a BIOSTAT® Aplus 5 L microbial bioreactor (Sartorius) containing 3 L of YCN medium supplemented with 400 g/L WXML. Seed cultures from the 30 h culture of yeast and the 24 h culture of *B. subtilis* were inoculated into the bioreactor with 5.0 % (v/v) inoculum. The medium pH was controlled at 6.0 using 25 % NaOH solution and 15 g/L of glucose was supplemented following the exhaustion of initial glucose from WXML. At the stage of micro-aerobic biotransformation, the conditions were set as temperature 30 °C, aeration 0.3 VVM and agitation 200 rpm. At the completion of biotransformation, the conditions were set as temperature 39 °C, aeration 1 VVM and agitation 300 rpm to allow for aerobic biotransformation. Samples were withdrawn periodically and centrifuged at 10,000*g* for 20 min for HPLC analysis. Medium glucose was also monitored offline using a GOD-POD kit (RSBIO, Shanghai, China) according to the manufacturer’s instructions.

In the case of scaled up experiments, the batch biotransformation was performed in 150 L and 30 m^3^ bioreactors following the same conditions as in the 5 L bioreactor with some modifications: the filling volume was 100 L and 20 m^3^, respectively; at the stage of micro-aerobic biotransformation, the aeration was set at 0.2 VVM and agitation at 60 rpm, and at the stage of aerobic transformation the parameters were 0.5 VVM and 100 rpm.

### Analytical methods

Dry cell weight (DCW) was measured using microbial cells from 50 mL of biotransformation solution. In brief, wet cell pellets were first washed twice using de-ionized water, centrifuged at 10,000*g* for 5 min to remove water, and dried at 105 °C to a constant weight. Analysis of sugar and sugar alcohols was performed on an HPLC system equipped with a Shodex RI 101 refractive index detector (RID). Prior to analysis, samples were centrifuged at 10,000*g* for 10 min, filtered through 0.22 μm syringe filters and diluted if necessary. Xylose, l-arabinose, glucose, galactose, xylitol and ethanol were separated on a prepacked analytical HPLC columns (Shodex SP0810, 8 × 30 mm, Pb^2+^ cation-exchange column) at 70 °C using distilled water as eluent at a flow rate of 1.0 mL/min. Analysis of furfural, HMF and their alcohols were performed using both HPLC and spectrophotometric method. In brief, 2 mL aliquots of the broth from the biotransformation experiment were reduced to dryness by lyophilization, and then the resulting materials were re-suspended in 1 mL of acetonitrile and filtered through a 0.45 μm nylon filter. Analysis was performed using reverse-phase HPLC equipped with an Eclipse XDB-C18 column (Bio-Rad, USA) set at 30 °C and a UV detector set at 280 nm. The mobile phase in this case consisted of acetonitrile: water (in v/v, 10 to 100 % in 15 min, 100 % for 10 min, 100 to 10 % in 2 min, and 10 % for 5 min) at a flow rate of 0.5 mL/min [24, 25, 39]. To determine the removal of furfural and HMF, measurement of absorbance at 280 nm (A_280_) was also adopted due to their peak absorbance at 280 nm [5, 16, 25, 40].
